# Quercetin, Catechin, and Diosmin as Modulators of Haloperidol–HSA Interactions: A Biophysical and Computational Study

**DOI:** 10.3390/ijms26146834

**Published:** 2025-07-16

**Authors:** Aleksandar Petrušić, Emina Mrkalić, Ratomir Jelić, Aleksandar Kočović, Miloš Milosavljević, Marko Antonijević, Miroslav Sovrlić

**Affiliations:** 1 Department of Social Pharmacy and Pharmaceutical Legislation, Faculty of Pharmacy, University of Belgrade, 11221 Belgrade, Serbia; 2 Department of Science, Institute for Information Technologies, University of Kragujevac, 34000 Kragujevac, Serbia; mantonijevic@uni.kg.ac.rs; 3 Department of Pharmacy, Faculty of Medical Sciences, University of Kragujevac, 34000 Kragujevac, Serbiasalekkg91@gmail.com (A.K.);; 4 Department of Pharmacology and Toxicology, Faculty of Medical Sciences, University of Kragujevac, 34000 Kragujevac, Serbia

**Keywords:** human serum albumin, flavonoids, haloperidol, interaction, fluorescence quenching, molecular docking, molecular dynamic

## Abstract

Potential interactions of haloperidol with food ingredients such as flavonoids may be of great importance both for understanding the pharmacokinetic interactions of xenobiotics with human serum albumin and for clinical practice itself. In this study, the effect of the flavonoids quercetin, catechin, and diosmin on the interaction of haloperidol and human serum albumin was examined. These flavonoids are very common in foods of plant origin. Haloperidol is a typical antipsychotic that has a pronounced binding affinity for human serum albumin. Fluorescence spectroscopy, molecular docking analysis, and molecular dynamics simulations were used for these tests. Previous studies have shown that all test substances bind to the same binding site on human serum albumin (Sudlow site I, Subdomain IIA). Fluorescence spectroscopy revealed that the tested flavonoids reduce the value of the haloperidol binding constant to human serum albumin (from 4.45 × 10^3^ in the binary system to 3.75 × 10^2^, 5.40 × 10^2^ and 6.24 × 10^2^ in the ternary systems, respectively), due to competition for the same binding site. Experimental results were confirmed by molecular docking analysis and molecular dynamics simulations.

## 1. Introduction

Human serum albumin (HSA) is one of the most abundant proteins in human blood. It has numerous functions, of which the maintenance of blood pH, maintenance of blood osmolarity, and transport role stand out [1]. HSA has great potential for binding and interacting with both endogenous and exogenous ligands. Of particular importance are pharmacokinetic interactions with numerous xenobiotics, where the binding of the drug to HSA plays a significant role in the redistribution of the drug to the free fraction and bound form, which later directly affects the duration of the drug effect and its effectiveness [2].

**Figure 1:**
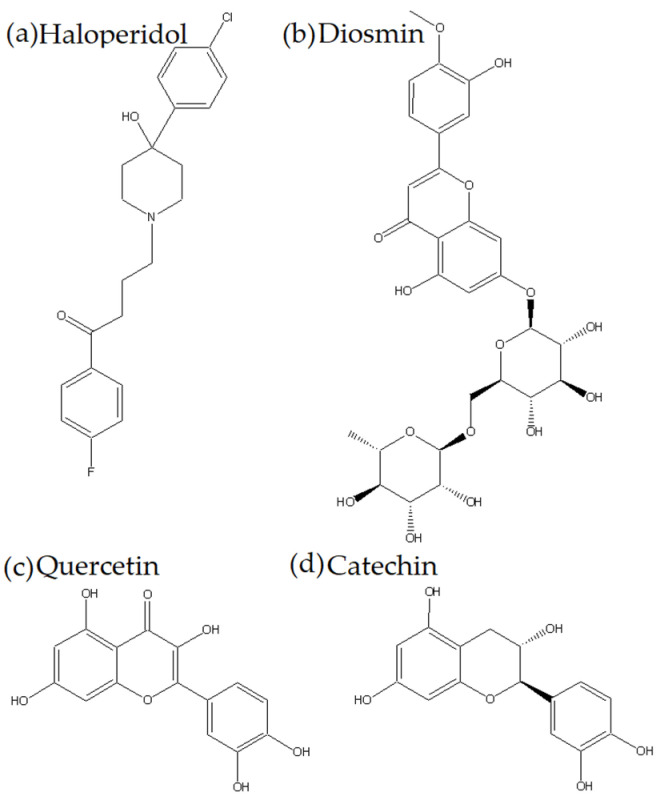
Chemical structures of investigated compounds (**a**): haloperidol—a typical antipsychotic; (**b**) diosmin—the flavone glycoside of diosmetin—exhibits antioxidant, anti-inflammatory, anti-breast cancer properties; (**c**) quercetin—a flavonoid from the flavonol group—exhibits antioxidant and anti-inflammatory properties; (**d**) catechin—a flavonoid from the flavan group—exhibits antimicrobial, antitumor, hepatoprotective, antidiabetic, and antioxidant effects.

Haloperidol (HPD) is a typical antipsychotic that chemically belongs to the group of butyrophenone antipsychotics (Figure 1). It works by blocking dopaminergic D2 receptors and, to a lesser extent, alpha-1 adrenergic receptors [3]. It is one of the most widely used drugs in the treatment of schizophrenia, bipolar disorder, and delirium [4]. The most important side effects are extrapyramidal motor side effects (akathisia, acute dystonia, tardive dyskinesia, parkinsonism), hyperprolactinemia, etc. [5]. Given the nature of the disease, the patients who take these medications, and the potential side effects, it is critical to understand how these drugs interact with food and other medications.

Flavonoids represent a diverse group of natural products with a phenolic structure [6]. They are very widespread in foods of plant origin that we consume every day [7]. The literature shows that the biological properties of flavonoids lead to their applications in many fields [8].

The pharmacological activities of flavonoids can be observed through their anti-inflammatory effects, as well as their roles in the treatment of antitumor and cardiovascular diseases, and other conditions [9,10].

Flavonoids with anti-inflammatory activity can be applied in the treatment of gastrointestinal tract diseases. As reported, baicalin, eupatilin, quercetin, diosmin, etc. demonstrated efficacy in the treatment of colitis [11,12,13,14].

Investigations into the antitumor properties of flavonoids have led to the conclusion that food rich with flavonoids has a preventive effect on colon cancer. Flavonoids can also lead to inhibition of tumor cell proliferation. Through experimental research, it has been shown that flavonoids have an impact on pancreatic cancer cells. Some flavonoids are effective in suppressing harmful changes such as proliferation, metastasis, and inflammation associated with lung cancer in A549 and H1299 lung cancer cell lines induced by nicotine [15].

Quercetin (QUE) is a flavonoid (Figure 1) from the group of flavonols that is very common in plants. It exhibits numerous biological activities that have been intensively investigated in recent years [13]. Due to its presence and activity, it is necessary to know well all its pharmacokinetic parameters, such as bioavailability, degree of binding to plasma proteins, and potential for interactions with other substances [16].

Catechin (CAT) is a polyphenolic compound (Figure 1) that belongs to the group of flavans, more precisely flavan-3-ols. Its effectiveness has been known since ancient times, and research has confirmed its antimicrobial, antitumor, hepatoprotective, antidiabetic, and antioxidant effects, among other effects [17].

Diosmin (DIO) is the flavone glycoside diosmetin (diosmetin 7-O-rutinoside) (Figure 1). It is most common in citrus species and is most often used in the treatment of vascular diseases (hemorrhoids, venous stasis, varicose veins, and venous ulcers) due to its antioxidant and phlebotonic effects [18,19,20].

The presence of flavonoids in the daily diet and their potential impact on human health indicate the importance of pharmacodynamic studies. Flavonoids are transported by the most abundant blood plasma protein, serum albumin (SA), through the formation of flavonoid-SA complexes [21]. Understanding flavonoid transport and the HSA binding mechanism could lead to new routes for the synthesis of flavonoid derivatives with improved biological characteristics.

The aim of this study was to examine the effect of quercetin, catechin, and diosmin on the interaction of haloperidol and human serum albumin by using spectroscopic methods, molecular docking analysis, and molecular docking simulations.

## 2. Results and Discussion

### 2.1. Fluorescence Quenching Measurements

Although HSA has three domains for binding endogenous and exogenous substances, drugs usually bind to two binding sites (Sudlow sites I and II), namely subdomains IIA and IIIA [22]. The presence of aromatic amino acids (Tyr, Trp, and Phe) in these subdomains is responsible for the fluorescence of the HSA molecule [23]. The intrinsic fluorescence of HSA is altered in the case of any change in the environment of the mentioned amino acids [24].

**Figure 2:**
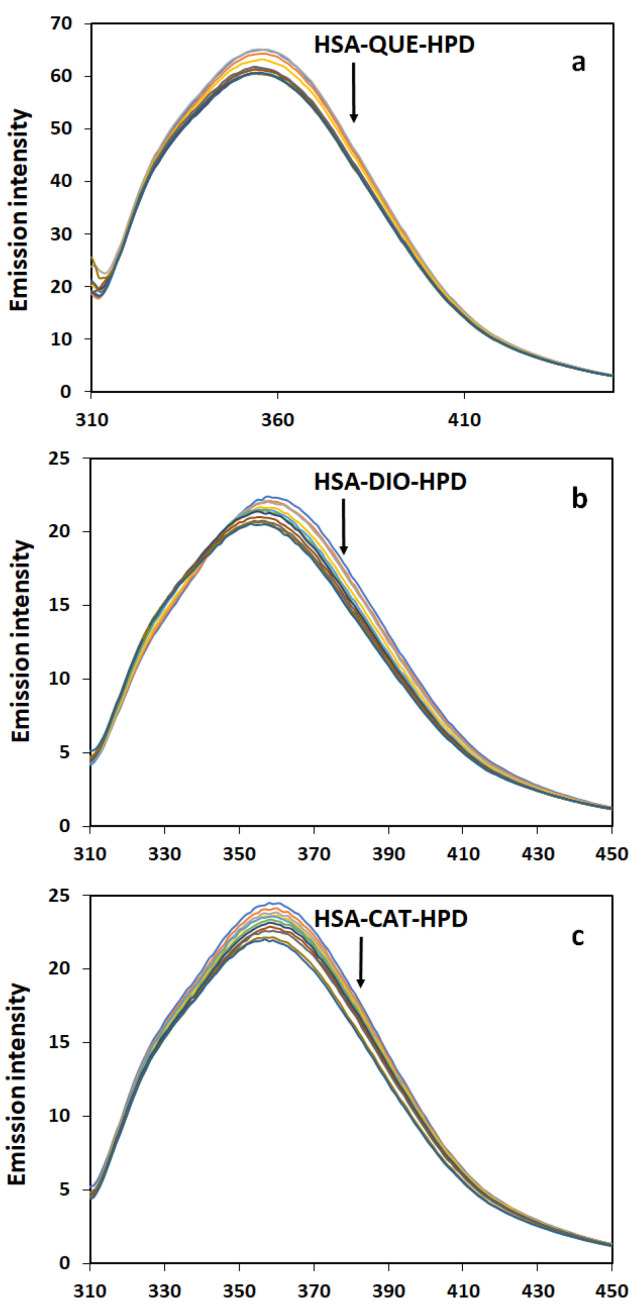
Fluorescence emission spectra of HSA-HPD in the presence of QUE (**a**), DIO (**b**), and CAT (**c**) (T = 303.15 K, pH = 7.4). [HSA] = 2 µM and [QUE] = 2 µM or [DIO] = 2 µM or [CAT] = 2 µM and [HPD] = 0 to 7.05 × 10^−5^ M.

In previous work, it has been shown that both haloperidol and the tested flavonoids (QUE, CAT, and DIO) bind to Sudlow site I, namely the IIA subdomain [25,26,27,28]. This means that there is competition for the same binding site among all tested substances. Figure 2 shows the quenching of fluorescence in ternary complexes of HSA and flavonoids at increasing HPD concentrations.

The intrinsic fluorescence of proteins can provide important information about their structure. At an excitation wavelength of 295 nm, which is characteristic for tryptophan, a strong fluorescence maximum peak can be observed at a wavelength of about 355 nm. As the HPD concentration increases, the intensity of internal fluorescence gradually decreases. This is characteristic for complex formation, in this case, a ternary system involving HSA-QUE-HPD, HSA-CAT-HPD, and HSA-DIO-HPD. The shape of the peaks and the wavelength of the maximum fluorescence are unchanged. The obtained results indicate that in binary HSA-HPD and in ternary has-flavonoid-HPD systems, the presence of the drug did not significantly cause a change in the microenvironment on the Trp residues in the HSA IIA subdomain.

### 2.2. Quenching Mechanism

The most often mentioned fluorescence quenching mechanisms are static and dynamic mechanisms. In addition, there is a possibility that within the interaction of two molecules, a combination of these two mechanisms will occur [29]. Dynamic quenching of fluorescence occurs in cases when there is a collision of molecules that interact, i.e., fluorophores and quenchers. On the other hand, static quenching of fluorescence is characteristic of cases when a non-fluorescent complex is formed between the quencher and the fluorophore [30]. There are several different ways to determine the mechanism of fluorescence quenching. In this paper, we used the Stern–Volmer equation (Equation (1)) to determine the mechanism of quenching the fluorescence of HSA in ternary complexes HSA-QUE-HPD, HSA-CAT-HPD, and HSA-DIO-HPD.

**Figure 3:**
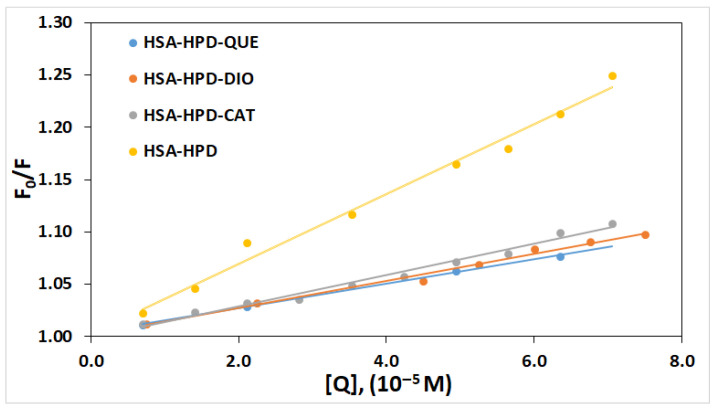
Stern–Volmer plots of the fluorescence quenching of HSA-HPD system by QUE, CAT, and DIO at 303.15 K.

The linear graph (Figure 3) indicates that only one fluorescence quenching mechanism (static or dynamic) is present. The values of quenching constants (*K*_q_) and Stern–Volmer constants (*K*_sv_) are presented in Table 1.

In the study involving the atypical antipsychotic olanzapine, the obtained Stern–Volmer graph is nonlinear, which indicates a combined mechanism of fluorescence quenching, especially at higher concentrations of the complex, and distinguishes it from haloperidol [31].

The *K*_SV_ value is about twice as high in the binary complex (HSA-HPD) than in the ternary HSA-QUE-HPD, HSA-CAT-HPD, and HSA-DIO-HPD complexes, which indicates that the examined flavonoids lead to a decreased interaction between HSA and HPD and thus to the formation of non-fluorescent complexes, which indicates that the quenching mechanism is static. Additional information on the fluorescence quenching mechanism is obtained based on the value of *K*_q_. If the *K*_q_ values are higher than the maximum scatter collision quenching constant of various quenchers with the biopolymers (2.0 × 10^10^ M^−1^ s^−1^) [32], it is considered that the quenching of the HSA fluorescence occurs as a consequence of the formation of the ground state complex and not as a consequence of dynamic collisions. In our study, the *K*_q_ values are higher than the cut-off value (2.0 × 10^10^ M^−1^ s^−1^), which additionally confirms that the mechanism of quenching the HSA fluorescence in the presence of QUE, CAT, and DIO and increasing HPD concentrations is static.

### 2.3. Binding Constant and Number of Binding Sites

Since HSA is one of the most abundant plasma proteins, the ability to bind exogenous substances to HSA is an important characteristic of substances that should be used as drugs. The binding constant of a substance, as well as the influence of other substances on it, greatly affects the pharmacokinetics and pharmacodynamics of drugs. Increasing the binding constant leads to a decrease in the free fraction of the drug, which reduces the pharmacological activity and at the same time increases the half-life, while reducing the binding constant increases the free fraction of the drug, increases the pharmacological effect, and can potentially lead to toxic effects. In addition, the half-life is reduced, and to some extent the distribution of the drug in the body is disturbed [1,2].

**Figure 4:**
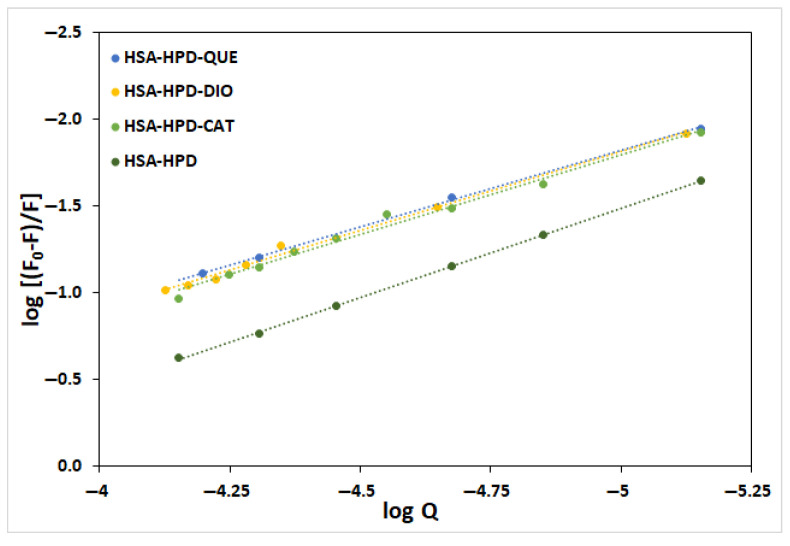
Logarithmic plots of the fluorescence quenching of HSA by HPD in the presence of QUE, CAT, and DIO at 303.15 K.

Figure 4 presents a diagram of the dependence of log (F_0_-F)/F against the concentration of complexes of HSA with QUE, CAT, and DIO in the absence of HPD, as well as in the presence of increasing concentrations of HPD. It can be seen from Table 1 that the number of binding sites for HPD to HSA is approximately one, regardless of the presence or absence of tested flavonoids. There is a slight decrease in these values, which indicates that there is competition in the binding of HPD and tested flavonoids to HSA [2]. This is to be expected given that all test substances have previously been reported to share a binding site (Sudlow site I). It also indicates that the possibility that the tested flavonoids and HPD bind simultaneously within the same binding site to the HSA. The values of the binding constant also indicate this option, as the value of the binding constant of ternary complexes decreases compared to the binary HSA-HPD complex. On the contrary, interactions between olanzapine and HSA in the presence and absence of CAT and DIO did not show a difference compared to the interactions of haloperidol-HSA in the presence of flavonoids (there was a decrease in *K*_b_ in the formation of ternary complexes HSA-CAT-OLZ and HSA-DIO-OLZ). This means that DIO and CAT showed competitive interference with HPD. However, in the case of QUE, an increase in *K*_b_ was observed, indicating that the QUE probably leads to allosteric modulation of the olanzapine binding site and enhanced binding to HSA [31]. It means that the presence of quercetin caused conformational changes in HSA and resulted in non-competitive interference in the presence of OLZ.

**Figure 5:**
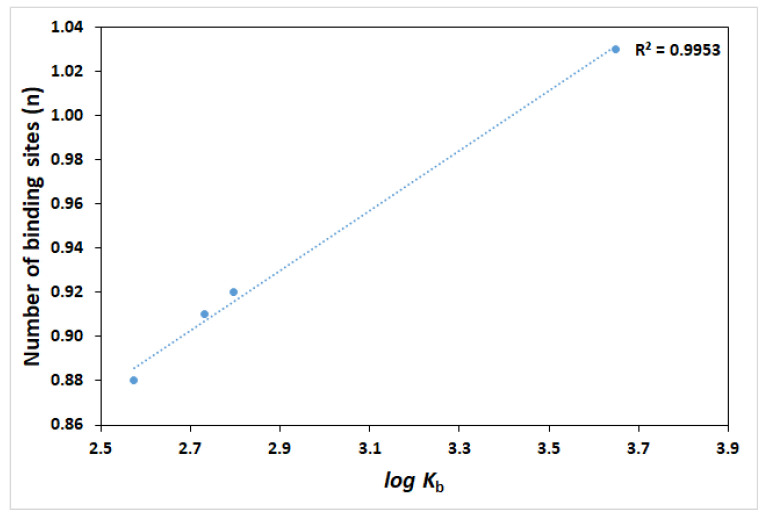
Relationship between the binding constants (log *K*_b_) and the number of binding sites (n) between HSA and HPD in the presence or absence of QUE, CAT, and DIO at 303.15 K.

The values of log *K*_b_ are proportional to the number of binding sites (n) with a high correlation coefficient (R^2^ = 0.9953) (Figure 5), which confirms that the mathematical model used (Equation (2)) is suitable for investigating the interactions between HPD and HSA [33].

### 2.4. Docking Analysis

**Figure 6:**
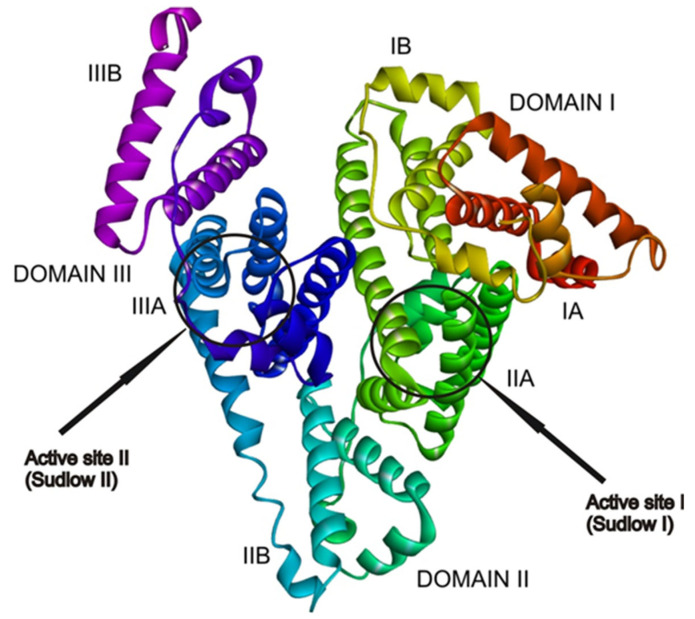
Active sites, domains and subdomains of HSA.

The HSA protein is able to bind molecules at six active binding sites [34,35,36]. In most cases, drugs bind to active sites I and II (Figure 6) because these sites have the highest binding affinities [33]. This is a consequence of the high amount of different amino acid residues that are forming hydrophobic cavities inside subdomains IIA and IIIA, where the active sites are located. At higher concentrations, drugs also bind to active sites with low binding affinity. The obtained experimental results in this paper show that the active site that has the highest affinity for binding the haloperidol is the same active site where flavonoid molecules are found, alongside other drugs such as warfarin and ibuprofen [25].

AGFR 1.0 software was employed to find the active site by configuring and computing affinity maps for the receptor molecule (Figure 6), which were further used in AutoDock 4.2. Docking results confirmed that the most favorable binding site in the receptor (Figure 6) for the investigated molecules is active site I (Sudlow I), located in domain II, subdomain IIA. This finding is in good agreement with the experimentally obtained results. The results presented in Table 2 include the binding free energie (ΔG*_bind_*) values, the inhibition constants (*K_i_*), and other thermodynamic parameters that describe the binding of the HPD molecule to HSA, as well as the binding of HPD to HSA-QUE, HSA-DIO, and HSA-CAT complexes.

**Figure 7:**
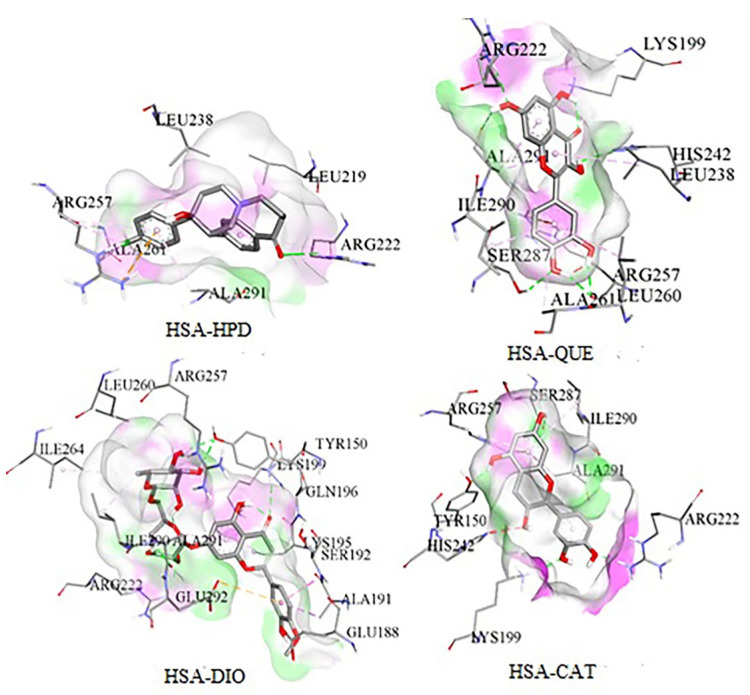
Interactions of HSA with the investigated compounds at Sudlow I active site, with the red surface indicating donors and green indicating acceptor, in hydrogen bonds.

As can be seen from the obtained results, the presence of the investigated compounds in blood will inhibit HSA-HPD bonding by a substantial amount. According to the inhibition constants in Table 2, the inhibition of HPD binding to HSA is highest in the presence of quercetin (QUE), followed by diosmin (DIO), and finally catechin (CAT). These results correlate well with the experimentally determined values. As can be seen from Figure 7, the inhibition of HPD binding emerges from the fact that the investigated flavonoids occupy the same active site of HSA as HPD. Because flavonoid ligands are voluminous, HPD is at least partially prevented from binding at active site I (Sudlow I). According to the results obtained via AGFR, HPD will bind at the active site in domain I, when active site Sudlow I is occupied by flavonoids. Because of that, differences in binding energies between pure HSA and has–flavonoid complexes with HPD are lower than expected, but the K_i_ value still indicates a good inhibitory potential.

Minor differences in binding energies can be attributed to structural similarities among the investigated compounds, as a large number of interactions with the protein are formed in favor of the aromatic structure of the flavonoid base of the QUE, CAT, and DIO (Figure 7). Therefore, the inhibition of HPD binding will occur in a similar way for all three investigated compounds. It should be noted that all the investigated compounds build interactions with the same amino acids, specifically those that are responsible for HPD binding (Figure 7).

The high degree of agreement between the experimentally observed decrease in haloperidol binding affinity (as indicated by reduced *K*_b_ values) and the increased inhibition constants (*K*_i_) from docking simulations supports the reliability of the molecular docking approach in predicting the impact of flavonoids. For example, the *K*_b_ value of the HSA-HPD binary complex decreases more than tenfold in the presence of quercetin, which correlates with a fourfold increase in *K*_i_. Such correlations reinforce the competitive binding mechanism proposed based on fluorescence quenching data.

Although all investigated flavonoids exhibited competitive binding at Sudlow site I, the relatively small differences in ΔG*_bind_* values suggest that steric hindrance and partial occupancy—rather than complete displacement—may govern the observed inhibition. This is particularly relevant for quercetin, which forms fewer hydrogen bonds but shows the highest inhibitory potential, implying that hydrophobic interactions dominate its mode of binding, whose stability will be further assessed in the specific timeframe via molecular dynamics simulations.

### 2.5. Molecular Dynamics

#### 2.5.1. Root Mean Square Deviation Analysis

**Figure 8:**
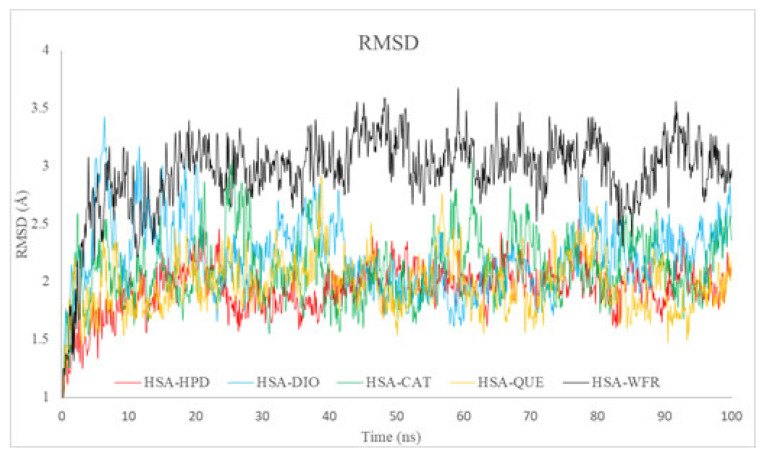
RMSD profiles for HSA complexes with haloperidol, diosmin, catechin, quercetin, and warfarin during 100 ns MD simulations.

The Root Mean Square Deviation (RMSD) was employed to assess the overall structural stability of the HSA complexes with the ligands under investigation (HPD, DIO, CAT, QUE) during the 100 ns simulations. All analyzed systems demonstrated relatively low RMSD values, ranging from approximately 1.5 to 3.5 Å, which suggests a high degree of structural stability (Figure 8). The HSA complexes with HPD, CAT, and QUE exhibited lower average RMSD values (~2 Å), indicating enhanced structural stability in comparison to DIO and WFR, which was used as standard drug for Sudlow I. The elevated RMSD observed for WFR indicates a more significant degree of conformational alterations or potential partial destabilization in comparison to the other ligands.

The results obtained are consistent with docking analyses, which demonstrated a notable binding affinity of flavonoids and HPD for Sudlow site I (subdomain IIA). Furthermore, the experimental fluorescence data demonstrated significant competitive binding interactions between HPD and flavonoids, especially QUE. This finding confirms that the ligands exhibit stable and competitive binding at the identical site on HSA.

#### 2.5.2. Root Mean Square Fluctuation Analysis

**Figure 9:**
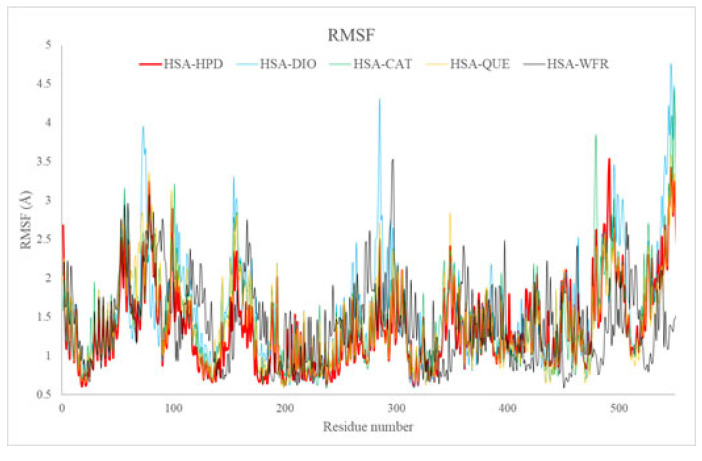
RMSF values of amino acid residues in HSA complexes with haloperidol, diosmin, catechin, quercetin, and warfarin.

The RMSF metric offers valuable insights into the flexibility of individual amino acids within HSA structures throughout simulation processes. The RMSF plots (Figure 9) exhibited comparable fluctuation profiles across all systems, revealing increased fluctuations in surface-exposed and terminal segments, which aligns with their intrinsic flexibility. Nonetheless, diminished fluctuations in areas surrounding the active binding site (subdomain IIA) were consistently noted for all ligands, providing clear evidence of stabilizing interactions with critical residues, including Tyr150, Lys199, Trp214, and Arg218.

The findings from the RMSF analysis corroborate the docking results, revealing substantial ligand interactions with critical residues at Sudlow site I. Moreover, the fluorescence experiments provided confirmation of stable binary complexes formed between flavonoids and HSA, which aligns with the results obtained from the RMSF analysis.

#### 2.5.3. Radius of Gyration Analysis

**Figure 10:**
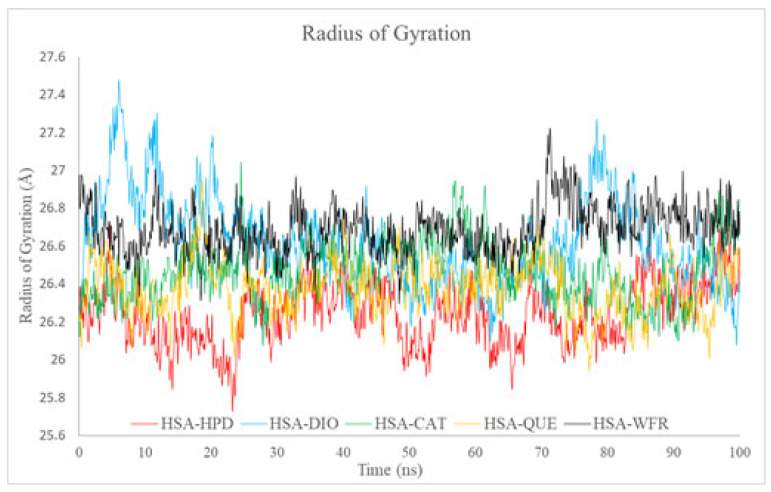
Radius of gyration (Rg) of HSA complexes with haloperidol, diosmin, catechin, quercetin, and warfarin over 100 ns of MD simulation.

Rg serves as a quantitative measure of the compactness of protein–ligand complexes during molecular dynamics simulations. All examined complexes exhibited stable Rg values, ranging from approximately 26.2 Å to 27.2 Å, with only minor fluctuations observed (Figure 10). The most compact complexes were identified for HSA-HPD and HSA-QUE, which aligns with the increased stability noted in the RMSD analysis. Conversely, the marginally elevated Rg values observed for the DIO and WFR complexes with HSA indicate a reduction in compactness of their conformations, accompanied by an enhancement in structural flexibility.

The results demonstrate the robust structural integrity of the protein when ligands are present, thereby supporting the docking findings that indicate significant affinity of ligands, notably QUE and HPD, for the compact structure of Sudlow site I. Experimental fluorescence data substantiate stable interactions, leading to a diminished HPD binding affinity when flavonoids, particularly QUE, are present, thereby reinforcing these findings.

#### 2.5.4. Analysis of the Number of Hydrogen Bonds (nHBs)

**Figure 11:**
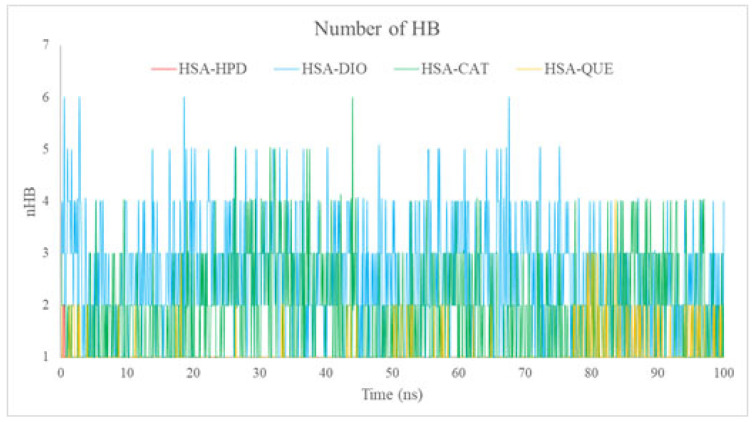
Number of hydrogen bonds (nHBs) formed between HSA and ligands (haloperidol, diosmin, catechin, quercetin) throughout the 100 ns MD simulations.

The nHB offers concrete evidence regarding the strength and durability of interactions between ligands and proteins. The complexes formed with DIO and CAT demonstrated the greatest frequency of hydrogen bonds during the simulations (Figure 11), typically ranging from 2 to 5. This phenomenon can be attributed to their hydroxyl-rich structures, which enhance the capacity for robust hydrogen bonding. The complexes formed with HPD and QUE exhibited a reduced number of stable hydrogen bonds, averaging between one to three bonds (Figure 11). The reduced number of hydrogen bonds identified for QUE, along with its notable stability, indicated by low RMSD and Rg values, implies that its binding stability is primarily attributable to hydrophobic interactions, which aligns with the findings from docking studies.

The experimental fluorescence data demonstrate a reduction in HPD binding constants when QUE is present. This observation supports the hypothesis that QUE occupies and obstructs the binding site primarily via hydrophobic interactions, along with a smaller number of consistent hydrogen bonds.

#### 2.5.5. Competitive Binding and the Role of Hydrophobic Interactions

To gain deeper insight into the competitive binding mechanism between HPD and flavonoids at Sudlow site I, we further analyzed the specific amino acid residues involved in ligand binding. Molecular docking and RMSF profiles from MD simulations consistently indicated the involvement of residues ALA261, LYS199, ARG222, ALA291, LEU219, and ARG257 in stabilizing both HPD and flavonoid interactions within Sudlow I active site. These residues are well-known contributors to Sudlow site I affinity and were consistently implicated in forming both hydrophobic contacts and hydrogen bonds with the ligands.

Among the flavonoids, QUE showed a distinctive binding profile. Despite forming fewer hydrogen bonds (1.05 on average) compared to diosmin (2.55) and catechin (1.85), it exhibited greater inhibition of HPD binding, as supported by the highest K_i_ value and the most pronounced reduction in K_b_. This observation, in conjunction with docking results showing modest HB contributions and MD-derived low RMSD and Rg values for HSA-QUE complexes, strongly suggests that hydrophobic interactions are the dominant stabilizing force in QUE binding. These findings highlight the crucial role of steric occupancy and hydrophobicity over hydrogen bonding in QUE’s competitive inhibition of HPD.

Such interplay of binding interactions emphasizes the complexity of flavonoid-induced modulation of drug–protein interactions and underscores the importance of residue-level analysis when evaluating pharmacokinetic competition at major plasma protein binding sites.

## 3. Materials and Methods

### 3.1. Chemicals and Reagents

Albumin from human serum (product No. A1887), haloperidol (Product No. H1512), catechin hydrate (product No. 22110), quercetin (product No. Q4951), diosmin (product No. D3525), and phosphate-buffered saline (Product No. P4417) were purchased from Merck KGaA, Darmstadt, Germany. All chemicals were used without further purification. Double-distilled water was used throughout the experiment.

### 3.2. Equipment and Spectral Measurements

All fluorescence spectra were recorded on an RF-1501 PC spectrofluorometer (Shimadzu, Kyoto, Japan) equipped with 1.0 cm quartz cells and a 150 W xenon lamp. An excitation wavelength of 295 nm was used. Fluorescence spectra were recorded at 303.15 K in the range of 310–450 nm. The widths of the excitation and emission slit widths were both fixed at 10 nm. The specified temperatures were controlled by a Julabo ED (v.2) open circulating bath (Julabo Labortechnik GmbH, Seelbach, Germany).

### 3.3. Preparation of Solutions

A PBS solution (0.01 M PBS, pH 7.4, 0.002.7 M KCI, 0.137 M NaCl) was prepared by dissolving the tablet in distilled water with control pH value of the solution. HSA (2 × 10^−5^ M) was dissolved in the PBS solution. The stock solution of HPD (3.2 × 10^−3^ M) was prepared by dissolving it in 5% methanol and then diluting it to 2.82 × 10^−4^ M with the PBS solution. The QUE, CAT, and DIO (2 × 10^−5^ M) stock solutions were dissolved in ethanol or a small amount of DMSO and diluted with the PBS solution. All solutions were made fresh and stored in the refrigerator at 4 °C prior to use.

### 3.4. Fluorescence Spectra of Haloperidol Binding with HSA in the Absence or Presence of Flavonoids

The fluorescence spectra were obtained using a constant HSA concentration (2.0 × 10^−6^ M) and by varying the HPD concentrations from 0 to 7.05 × 10^−5^ M (7.05 × 10^−6^ M, 1.41 × 10^−5^ M, 2.12 × 10^−6^ M, 2.82 × 10^−5^ M, 3.53 × 10^−5^ M, 4.23 × 10^−5^ M, 4.94 × 10^−5^ M, 5.64 × 10^−5^ M, 6.35 × 10^−5^ M and 7.05 × 10^−5^ M). Two hundred microliters of the HSA solution and 200 μL of flavonoid solution were added to 5 mL flasks, respectively. After a while, appropriate amounts of 3 × 10^−4^ M HPD were added and then diluted with the PBS solution. The concentration of flavonoids was 2·× 10^−6^ M which was the same as that of HSA (1:1). The resultant mixtures were then incubated at 303.15 K for 1.0 h. The fluorescence emission spectra were scanned in the range of 310–450 nm.

### 3.5. Molecular Docking

In order to examine the influence of different flavonoid molecules on the binding of haloperidol (HPD) to human serum albumin (HSA), molecular docking simulations were performed. The active site of HSA was determined by the AutoGridFR (AGFR) program [37]. The geometries of the investigated compounds were optimized, and energy minima were found using the Gaussian 09 software package with density functional theory (DFT), employing the B3LYP functional with 6–311++G(d,p) [38,39,40]. The crystal structure of HSA was obtained from the Brookhaven Protein Data Bank (PDB ID:1HK1 [41]). Protein was prepared for docking in Discovery Studio 4.0 [42]. Polar hydrogens, Kollman charges, and other parameters were added using the graphical interface from AutoDockTools (ADT) [43]. AutoDock 4.2 software was used for molecular docking simulations. The Lamarckian Genetic Algorithm (LGA) was implemented for protein–ligand and complex–ligand flexible docking. The parameters for the LGA method were determined as follows: the maximum number of energy evaluations was 250,000, the maximum number of generations was 27,000, and the mutation and crossover rates were 0.02 and 0.8, respectively. The algorithms that AutoDock 4.2 software is based on can predict ligand positions within the protein target and assess them by scoring functions defined by setting the grid box. A grid box with dimensions 56 × 50 × 58 Å^3^ in -*x*, -*y*, and -*z* directions of has, with the center at x = −5.659, y = 1.623, z = 10.844, was used to cover the protein binding site and accommodate the ligand to move freely. A grid point spacing of 0.375 Å was used for auto grid runs. The center of the small molecule was set at coordinates x = −0.1406, y = −0.0465, z = 0.0991. For each ligand, docking was performed using 50 genetic algorithm runs, yielding 50 binding poses per ligand, from which the lowest-energy conformation was selected for further analysis. The obtained results were analyzed and illustrated in Discovery Studio 4.0.

### 3.6. Molecular Dynamics Simulations

Molecular dynamics (MD) simulations of human serum albumin complexes with the investigated ligands (haloperidol, diosmin, catechin, quercetin, and warfarin—which serves as the standard for Sudlow I) were conducted to evaluate their structural stability and binding interactions at the molecular level. The initial structures of ligand–protein complexes were generated utilizing the most stable conformations derived from molecular docking simulations. The crystal structure of HSA, identified by PDB ID: 1HK1, was obtained from the Protein Data Bank (PDB) [41].

The CHARMM-GUI webserver [44,45] was employed for the preparation of simulation inputs, utilizing the Solution Builder module to explicitly solvate each system in a TIP3P water box, and ions were added to neutralize the net system charge and reach a physiological ionic strength of 0.15 M. A total of 136 K^+^ and 123 Cl^−^ ions were added accordingly. Protonation states of titratable residues were assigned based on local microenvironments. In particular, Glu244 was protonated (GLUP), and Asp13 was protonated (ASPP), while all other residues were kept in their standard protonation forms as assigned by CHARMM default settings. The parameterization of the protein–ligand complexes was conducted utilizing the AmberFF14SB [46] for the protein atoms and the General AMBER Force Field (GAFF) [47] for the ligands. The generation of ligand parameters was accomplished through the AM1-BCC method [48], as implemented in Antechamber from AmberTools22 [49].

MD simulations were performed utilizing AMBER22 software suite [50]. Every system was subjected to a systematic equilibration protocol that included minimization, heating, and equilibration phases. The initial energy minimization process was conducted in two distinct stages: Two thousand five hundred steps of steepest descent were executed, succeeded by two thousand five hundred steps utilizing the conjugate gradient algorithm, with harmonic restraints of 10 kcal mol^−1^ Å^−2^ imposed on both protein and ligand atoms. The system was subsequently subjected to a gradual heating process, transitioning from 0 to 300 K throughout 500 ps within the canonical (NVT) ensemble, while applying harmonic restraints on both the protein and ligand. The subsequent phase involved a 1 ns equilibration at 300 K under isothermal–isobaric (NPT) conditions at 1 atm pressure. This was achieved through the application of a Langevin thermostat [44] and a Berendsen barostat [51], which effectively maintained constant temperature and pressure, respectively. The restraints were systematically diminished and ultimately eliminated.

Production molecular dynamics simulations were conducted under NPT ensemble conditions at a temperature of 300 K and a pressure of 1 atm for a duration of 100 ns for each complex. The particle mesh Ewald (PME) method [52] was employed to compute long-range electrostatic interactions, whereas van der Waals interactions were truncated at a cutoff of 10 Å. The SHAKE algorithm [53] imposes constraints on hydrogen-containing bonds, thereby facilitating an integration time step of 2 fs. Trajectories were recorded at intervals of 10 picoseconds for further analysis.

The trajectory analysis encompassed the computation of Root Mean Square Deviation (RMSD), Root Mean Square Fluctuation (RMSF), radius of gyration (Rg), and the quantification of hydrogen bonds, utilizing CPPTRAJ v6.4.4. software from AmberTools22 [34]. The analyses conducted evaluated the structural stability and interactions between ligands and proteins, as well as the conformational behavior observed throughout the simulation. To evaluate the robustness and reproducibility of the observed protein–ligand interactions, we conducted MD simulations in triplicate, each lasting 100 ns, for all studied systems. This approach allowed us to confirm the consistency of structural behavior across independent runs. The results demonstrated high concordance in RMSD, Rg, and the nHB among replicas, with only minor fluctuations indicative of stable binding.

Detailed comparisons of RMSD, Rg, and nHB values across all replicas are provided in the Supplementary Information (Figures S1–S4). These results strongly support the conclusion that the tested flavonoids persistently occupy Sudlow site I and compete with haloperidol in a consistent and reproducible manner throughout the simulations.

### 3.7. Data Analysis

The fluorescence quenching mechanism was determined according to the Stern–Volmer Equation (1) [54,55,56,57]

F_0_/F = 1 + *k*_q_τ_0_[Q] = 1 + *K*_SV_ [Q] (1)

where F_0_ and F are fluorescence intensities of HSA in the absence and presence of the quencher, respectively; k_q_ and τ_0_ are the quenching rate constant and average lifetime [10^−8^ s^−1^] in the absence of the drug, respectively; [Q] is the concentration of the quencher, and *K*_SV_ is the Stern–Volmer quenching constant [Lmol^−1^]. *K*_SV_ was determined as the linear regression of a plot of F_0_/F against [Q].

The binding constant (*K*_b_) and the number of binding sites of protein (n) are determined according to the equation [58] shown below:

log (F_0_−F)/F = log*K*_b_ + nlog[Q] (2)

The values of K_b_ and n were obtained from the intercept and slope of the plots of log (F_0_−F)/F versus log [Q].

## 4. Conclusions

The effect of quercetin, catechin, and diosmin on the interaction of haloperidol with human serum albumin was investigated in this paper. Fluorescence spectroscopic studies have shown that during the formation of ternary complexes of human serum albumin, haloperidol, and flavonoids, fluorescence is quenched, most likely by a static mechanism. The binding constant also decreases, which can lead to an increase in the free fraction of haloperidol in the serum, an enhanced effect, and a more frequent occurrence of side effects. Molecular docking analysis is in accordance with experimentally obtained data. It has been shown that all test substances bind to the same binding site (Sudlow site I, Subdomain IIa), and that two molecules can bind simultaneously, with the mechanism of inhibition of haloperidol binding to human serum albumin being similar to that observed for all test molecules.

The results from MD simulations, including RMSD, RMSF, Rg, and nHB, provide strong support for the experimental and docking findings. All complexes exhibited clear structural stability, with particular emphasis on the HSA-HPD and HSA–flavonoid complexes, namely QUE, CAT, and DIO. Flavonoids competitively occupied Sudlow site I, thereby stabilizing it and inhibiting the binding of HPD, a finding that was directly confirmed through fluorescence experiments and molecular docking analyses. The results obtained are crucial for comprehending the pharmacokinetic interactions that occur between dietary flavonoids and medications such as HPD, emphasizing possible changes in therapeutic efficacy and pharmacodynamics.

These findings are of great importance both because of the presence of the examined flavonoids in food and because of the type of disease for which haloperidol is used. Any fluctuation in the concentration of haloperidol in the patient’s serum can lead to a change in the effectiveness of the therapy and a possible worsening of the condition in patients undergoing this therapy.

## Figures and Tables

**Table 1:** Binding parameters of binary (HSA-HPD) and ternary (HSA-QUE-HPD, HSA-DIO-HPD, HSA-CAT-HPD) systems at 303.15 K.

System	*K*_SV_/dm^3^ mol^−1^	*K*_q_/dm^3^ mol^−1^ s	R^2^ *	*K*_b_/dm^3^ mol^−1^	n	R^2^
HSA-HPD	3.35 × 10^3^	3.37 × 10^11^	0.9869	4.45 × 10^3^	1.03	0.9997
HSA-QUE-HPD	1.18 × 10^3^	1.18 × 10^11^	0.9988	3.75 × 10^2^	0.88	0.9992
HSA-DIO-HPD	1.30 × 10^3^	1.30 × 10^11^	0.9881	5.40 × 10^2^	0.91	0.9933
HSA-CAT-HPD	1.50 × 10^3^	1.50 × 10^11^	0.9841	6.24 × 10^2^	0.92	0.9861

* correlation coefficient.

**Table 2:** Estimated values of binding energies for HSA-HPD and HSA-QUE, HSA-DIO, and HSA-CAT complexes with HPD.

HSA-Ligand Complex	ΔG_*bind*_ kJ/mol	*K*_i_ (nM)	ΔG_*inter*_ kJ/mol	ΔG_*vdw+hbond+desolv*_ kJ/mol	ΔG_*elec*_ kJ/mol	ΔG_*total*_ kJ/mol	ΔG_*tor*_ kJ/mol
HSA-HPD	−40.7	75.6	−43.2	−42.8	−0.4	−6.2	8.7
HSA-QUE-HPD	−37.0	325.6	−38.2	−37.9	−0.2	−7.6	8.7
HSA-DIO-HPD	−37.2	298.4	−41.5	−41.4	−0.1	−4.5	8.7
HSA-CAT-HPD	−38.0	221.5	−42.5	−42.0	−0.5	−4.3	8.7

R^2^—correlation coefficient; *K*_i_ values were estimated by AutoDock using internal scoring functions that include torsional entropy and empirical corrections and may slightly differ from the theoretical values derived directly from ΔG*_bind_*.

## Data Availability

The data used in this study are available upon request from the corresponding author.

## Supplementary Materials

The following supporting information can be downloaded at: https://www.mdpi.com/article/10.3390/ijms26146834/s1.

## Abbreviations

The following abbreviations are used in this manuscript:

HSA	Human serum albumin
HPD	Haloperidol
QUE	Quercetin
DIO	Diosmin
CAT	Catechin
MD	Molecular dynamics
RMSD	Root Mean Square Deviation
RMSF	Root Mean Square Fluctuation
