# Supplementary material for: Quercetin, Catechin, and Diosmin as Modulators of Haloperidol–HSA Interactions: A Biophysical and Computational Study
Source: Int J Mol Sci. 2025 Jul 16;26(14):6834. doi: 10.3390/ijms26146834 (PMC12295124; doi:10.3390/ijms26146834)
Supplement: Supplementary file 1 [file ijms-26-06834-s001.zip › ijms-3736711-supplementary.pdf]

Supplementary Information

Quercetin, Catechin, and Diosmin as Modulators of Haloperidol–HSA Interactions: A Biophysical and Computational Study

Aleksandar Petrušić[1], Emina Mrkalić [2,*] Ratomir Jelić[3], Aleksandar Kočović[3], Miloš Milosavljević[4], Marko Antonijević[2], Miroslav Sovrlić[3]

[1]Department of Social Pharmacy and Pharmaceutical Legislation, Faculty of Pharmacy, University of Belgrade, 11221 Belgrade, Serbia; hadzipetrusic@icloud.com
[2]Department of Science, Institute for Information Technologies, University of Kragujevac, 34000 Kragujevac, Serbia; emina.mrkalic@pmf.kg.ac.rs
[3]Department of Pharmacy, Faculty of Medical Sciences, University of Kragujevac, 34000 Kragujevac, Serbia; sofke-ph@hotmail.com
[4]Department of Pharmacology and toxicology, Faculty of Medical Sciences, University of Kragujevac, 34000 Kragujevac, Serbia; milosavljevicmilos91@gmail.com
*Correspondence emina.mrkalic@pmf.kg.ac.rs

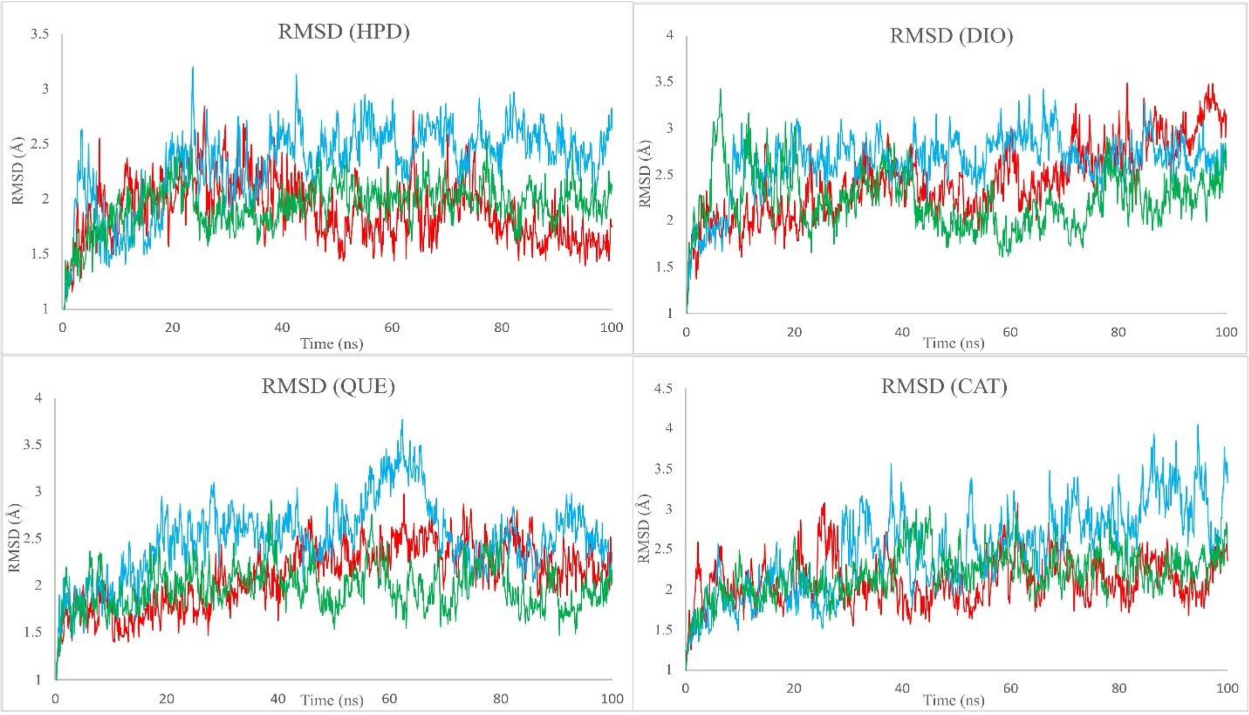

Figure S1. Root Mean Square Deviation (RMSD) profiles for three independent 100 ns MD simulations of investigated complexes.

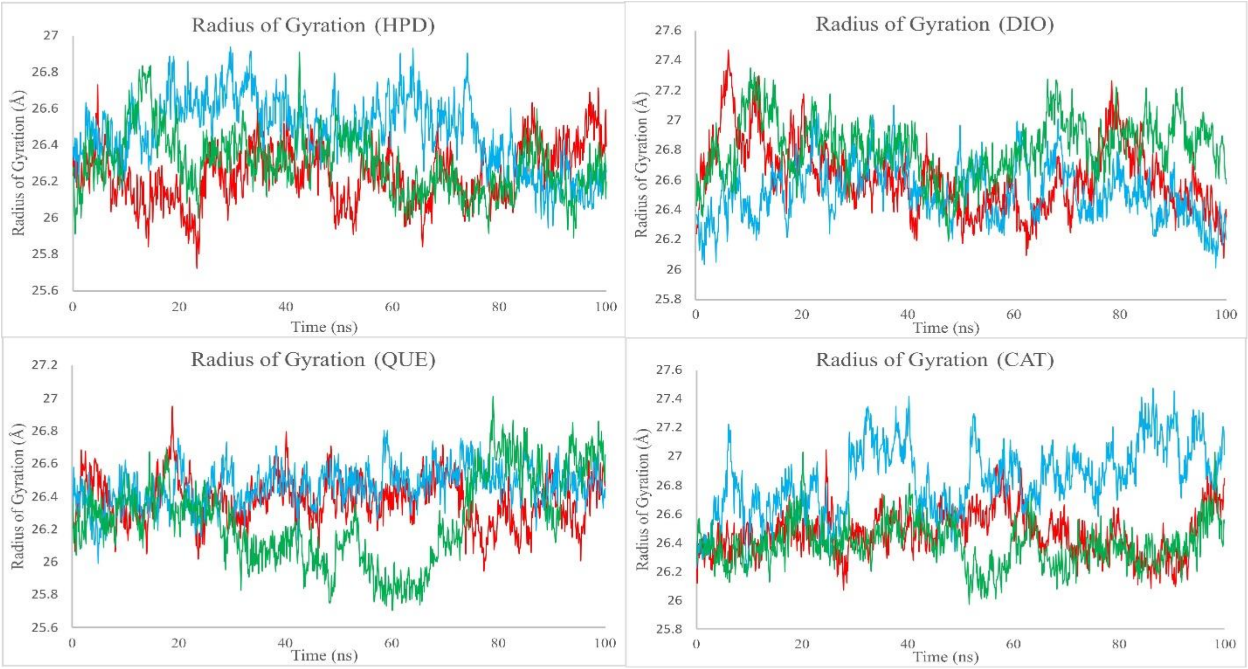

Figure S2. Radius of Gyration (Rg) profiles for three independent 100 ns MD simulations of investigated complexes.

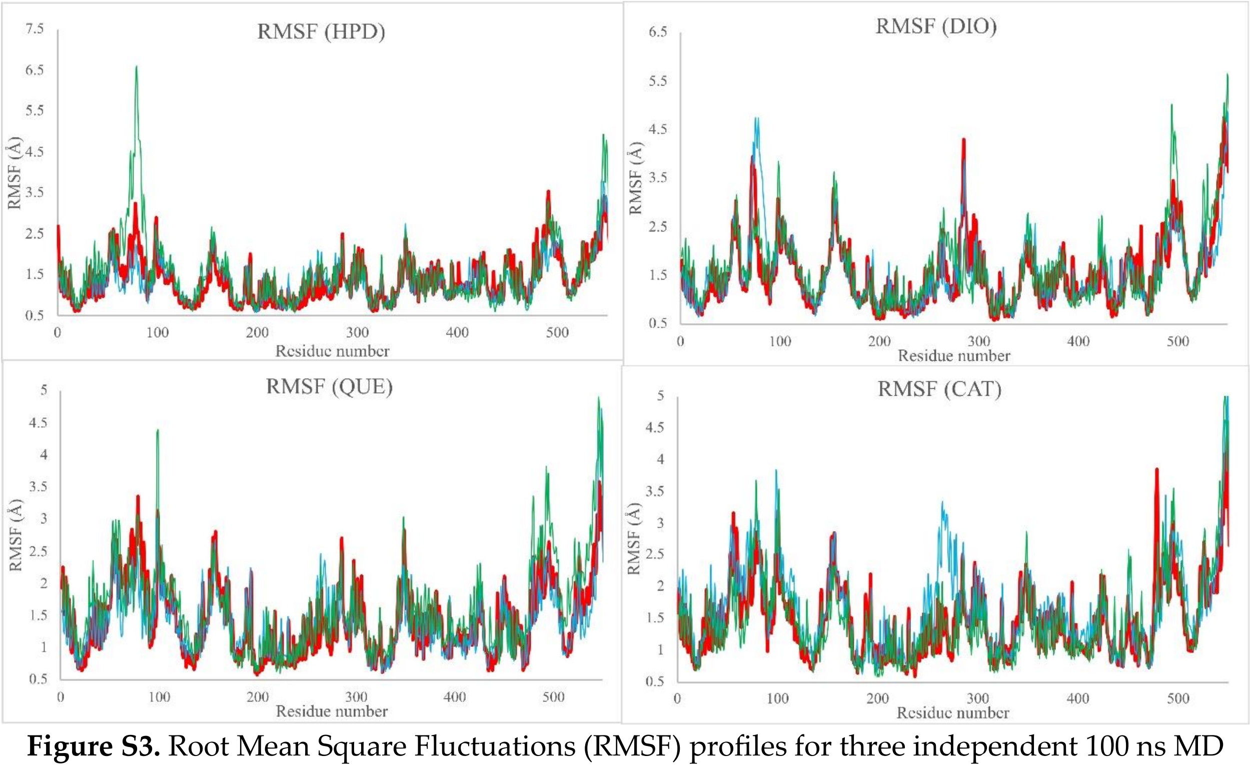

Figure S3. Root Mean Square Fluctuations (RMSF) profiles for three independent 100 ns MD simulations of investigated complexes.

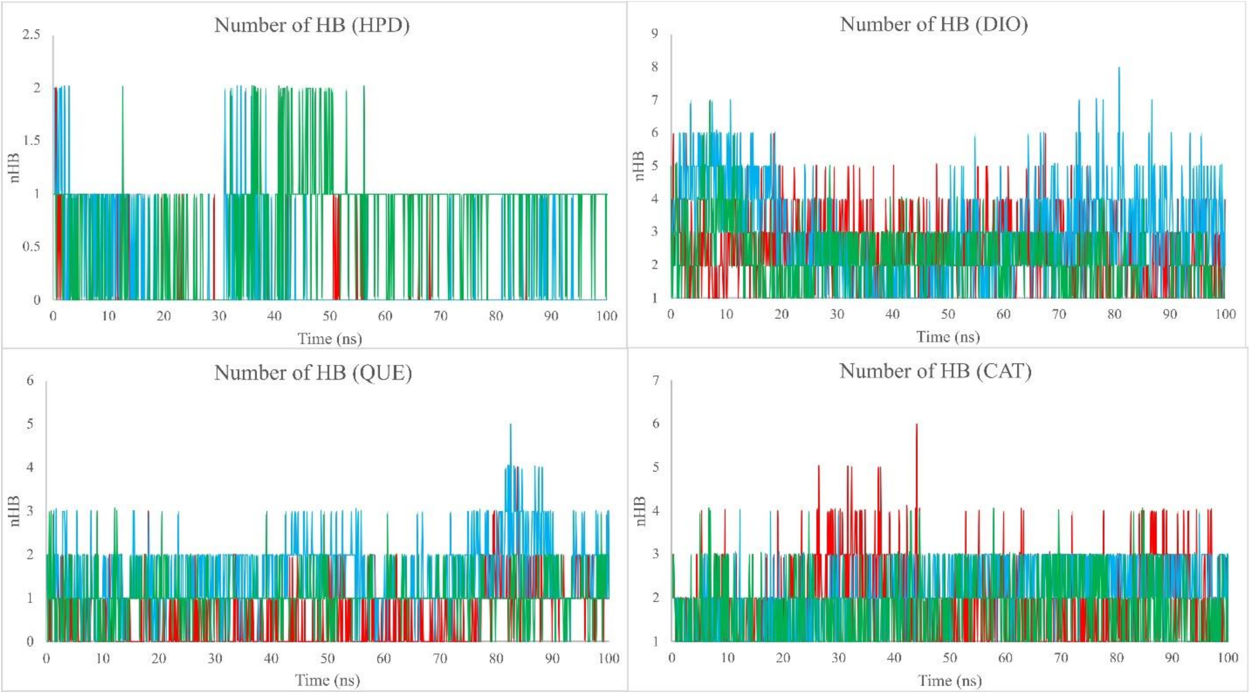

Figure S4. Number of Hydrogen Bonds (nHB) profiles for three independent 100 ns MD simulations of investigated complexes.
